# Ergogenic effects of caffeine and sodium bicarbonate supplementation on intermittent exercise performance preceded by intense arm cranking exercise

**DOI:** 10.1186/s12970-015-0075-x

**Published:** 2015-02-27

**Authors:** Matthaus Marriott, Peter Krustrup, Magni Mohr

**Affiliations:** Sport and Health Sciences, College of Life and Environmental Sciences, St. Luke’s Campus, University of Exeter, Exeter, UK; Department of Nutrition, Exercise and Sports, Section of Human Physiology, Copenhagen Centre for Team Sport and Health, University of Copenhagen, Copenhagen, Denmark; Faculty of Natural and Health Sciences, University of the Faroe Islands, Jónas Broncks gøta 25. 3rd floor, Tórshavn, Faroe Islands; Center of Health and Human Performance, Department of Food and Nutrition, and Sport Science, University of Gothenburg, Gothenburg, Sweden

**Keywords:** Yo-Yo IR2 test performance, Fatigue, Blood lactate, Rating of perceived exertion, Team sport athletes

## Abstract

**Background:**

Caffeine and sodium bicarbonate ingestion have been suggested to improve high-intensity intermittent exercise, but it is unclear if these ergogenic substances affect performance under provoked metabolic acidification. To study the effects of caffeine and sodium bicarbonate on intense intermittent exercise performance and metabolic markers under exercise-induced acidification, intense arm-cranking exercise was performed prior to intense intermittent running after intake of placebo, caffeine and sodium bicarbonate.

**Methods:**

Male team-sports athletes (n = 12) ingested sodium bicarbonate (NaHCO_3_; 0.4 g.kg^−1^ b.w.), caffeine (CAF; 6 mg.kg^−1^ b.w.) or placebo (PLA) on three different occasions. Thereafter, participants engaged in intense arm exercise prior to the Yo-Yo intermittent recovery test level-2 (Yo-Yo IR2). Heart rate, blood lactate and glucose as well as rating of perceived exertion (RPE) were determined during the protocol.

**Results:**

CAF and NaHCO_3_ elicited a 14 and 23% improvement (*P* < 0.05), respectively, in Yo-Yo IR2 performance, post arm exercise compared to PLA. The NaHCO_3_ trial displayed higher [blood lactate] (*P* < 0.05) compared to CAF and PLA (10.5 ± 1.9 vs. 8.8 ± 1.7 and 7.7 ± 2.0 mmol.L^−1^, respectively) after the Yo-Yo IR2. At exhaustion CAF demonstrated higher (*P* < 0.05) [blood glucose] compared to PLA and NaHCO_3_ (5.5 ± 0.7 vs. 4.2 ± 0.9 vs. 4.1 ± 0.9 mmol.L^−1^, respectively). RPE was lower (*P* < 0.05) during the Yo-Yo IR2 test in the NaHCO_3_ trial in comparison to CAF and PLA, while no difference in heart rate was observed between trials.

**Conclusions:**

Caffeine and sodium bicarbonate administration improved Yo-Yo IR2 performance and lowered perceived exertion after intense arm cranking exercise, with greater overall effects of sodium bicarbonate intake.

## Introduction

Fatigue during high-intensity intermittent exercise is complex and multifaceted. Early speculation regarding the aetiology of fatigue commends that high rate of lactic acid production and a concomitant fall in blood and muscle pH [1], which may have multiple indirect and direct impairing effects on centrally and peripherally mediated fatigue-resistance. Caffeine and sodium bicarbonate (NaHCO_3_) are two supplements frequently consumed to elicit ergogenic effects on high-intensity exercise performance [2].

Effects of caffeine on intense intermittent exercise performance have for example been studied by Stuart et al. [3] demonstrating improved repeated sprint performance during a simulated rugby game trial. Moreover, team-sport athletes improved both total work and mean power output during an intermittent cycle sprint protocol [4] and Yo-Yo IR2 performance by 16% after caffeine intake [5]. However, Glaister et al. [6] showed that although the fastest sprint time in a repeated sprint test was observed with caffeine intake, the magnitude of fatigue in the caffeine condition appeared to be greater compared to a placebo trial. Furthermore, no ergogenic effect has been found on sprint performance during the Loughborough intermittent shuttle test [7]. Thus, the effects of caffeine intake on intense intermittent exercise protocols are equivocal.

NaHCO_3_ ingested 90–150 min prior exercise has been used as an ergogenic aid for athletic events highly dependent on anaerobic glycolysis, since the ergogenic potential that NaHCO_3_ might elicit is suggested to depend upon the demands of the activity being sufficient to induce performance inhibiting levels of metabolic acidosis [8]. NaHCO_3_ ingestion has been reported to improve competitive and laboratory-based protocols lasting 1–7 min including swimming, middle distance running, rowing and repeated sprinting [9]. In addition, improvement in performance during a repeated sprint protocol is reported [10]. However, other studies are less affirmative and demonstrating no performance enhancing effects on high-intensity intermittent cycling [11]. Further discrepancies have been illustrated by Cameron et al. [12] whereby no benefits were observed during a high-intensity rugby-specific training session followed by a repeated-sprint test. The absence of effects within the aforementioned studies is potentially due to insufficient metabolic taxation.

The Yo-Yo Intermittent Recovery test level 2 (Yo-Yo IR2) consists of 20-m shuttle runs at progressive running speed and has a high anaerobic energy turnover [13]. Thus, in order to examine the effect of caffeine and NaHCO_3_ intake on high-intensity intermittent exercise the Yo-Yo IR2 test can be utilized. Moreover, engaging in intense arm exercise prior to repeated high-intensity running elevates the levels of leg muscle and blood [lactate] and [H^+^] [14], as well as increasing the accumulation rate in muscle interstitial [K^+^] resulting in decreased knee extensor exercise performance [15]. Therefore, intense upper-body exercise prior to running exercise can be applied to induce pre-exercise muscle acidosis and high metabolic disturbance without exercising the legs.

Thus, the aim of the present study was therefore to compare the effects of caffeine and NaHCO_3_ supplementation 70–90 min prior to exercise, respectively, on Yo-Yo IR2 performance and physiological response to intense intermittent exercise with prior metabolic acidosis induced by intense arm cranking exercise.

## Methods

### Participants

Twelve healthy male participants involved in sub-elite team-sports (age: 20.8 ± 1.4 (±SD) yrs.; height: 183 ± 7 cm; body mass: 78.9 ± 5.4 kg) volunteered to participate in this study. Participants gave their written informed consent to participate prior to the experimental procedures and the study conforms the ethical guidelines of the Declaration of Helsinki. The study was approved by the University of Exeter Ethics Committee.

### Design

The participants reported to the laboratory on five separate occasions with at least four days between visits. On the initial visit to the laboratory the participants were familiarised to the arm exercise protocol conducted on an upper body arm cranking ergometer (Lode BV, Angio, Netherlands) to determine individual specific power outputs as previously described [15]. On the second occasion participants performed a baseline, control (CON) Yo-Yo IR2 that they were familiarized to prior to the study [13]. The CON-trial was performed without any supplementation or prior arm-exercise. Participants were then assigned in a single-blind, randomized, crossover design to receive placebo (PLA; plain flour), caffeine (CAF) or sodium bicarbonate (NaHCO_3_) supplementation.

### Experimental procedure

On each experimental visit, participants were asked to report to the laboratory 100 min prior to the initiation of the Yo-Yo IR2 in a fully hydrated state and ≥2 h postprandial. The tests were carried out at the same time of the day (±1 h). The participants were not permitted to consumed alcohol 24 h prior to testing or to take any other form of dietary supplements for the duration of the study and to avoid strenuous exercise 24 h preceding each experimental trial. In addition the participants were asked to avoid food items containing caffeine prior to the experimental days. Moreover, the food intake was noted the day prior to the first test trial and replicated prior to the remaining trials. Upon arrival to the laboratory participants were fitted with a heart rate (HR) monitor (Polar Electro, Kempele, Finland) and a baseline fingertip capillary blood sample was obtained. Participants were then given either CAF or PLA (blinded) or NaHCO_3_ to orally ingest under the supervision of the researchers. 70 min before the start of the Yo-Yo IR2 either CAF or PLA was taken orally in gelatine capsule (6 mg.kg^−1^ body mass; 474 ± 31 mg; see Mohr et al. [5]). NaHCO_3_ was ingested orally in 21–25 gelatine capsules (0.4 g.kg^−1^ body mass; 31.6 ± 1.6 g) and supplementation began 90 min prior to the start of the Yo-Yo IR2 with 7 ml.kg^−1^ of water drank ad libitum over a 30 min period as described by Carr et al. [9]. The respective NaHCO_3_ intake protocol has in a pilot study been demonstrated to markedly raise the blood HCO^−^_3_ concentration (data not shown).

The arm exercise protocol lasted for 17 min, during which the participants maintained a constant cadence (80 ± 5 RPM) at individualised power outputs (157.6 ± 7.4 W). The protocol, adapted from others [14,15] consisting of 4×1-min and 1×1.5-min exercise periods separated by 0.5-min recovery, followed by 4.5 min of recovery and a final 1-min exercise period. Immediately after completion of the arm exercise, a blood sample was taken. The Yo-Yo IR2 began 4 min after the completion of the arm exercise protocol corresponding to 70 min post CAF and PLA intake and 90 min after the NaHCO_3_ ingestion. The Yo-Yo IR2 test was completed on a wooden surface and consists of repeated two 20-m runs at a progressively increased speed controlled by audio bleeps from a CD player [13]. The test-leader controlling the Yo-Yo IR2 test was blinded in relation to drug-treatment.

Fingertip capillary 300 μL blood samples were collected in heparin-fluoride coated Microvette CB 300 tubes (Sarstedt Ltd, UK) which were immediately stored on ice and subsequently analysed to determine blood [lactate] and [glucose] after the protocol [5] using a YSI 2500 Lactate Analyser (YSI, Yellow Springs, US) having a test-retest coefficient of variance of <2%. Blood samples were obtained: prior to supplementation on arrival to the laboratory, immediately after arm exercise, immediately after Yo-Yo IR2 exhaustion, 1, 3 and 5 min post exhaustion. Heart rate was determined during the entire protocol while rating of perceived exertion (RPE) values using the Borg scale [16] were recorded during the Yo-Yo IR2 at 160, 280, 440, 600 m and at exhaustion.

### Statistical analyses

Differences between baseline Yo-Yo IR2 performance and PLA Yo-Yo IR2 after arm exercise were analysed using a paired-samples *t*-test. A one-way repeated measures ANOVA was used to determine the influence of supplementation on the performance in the Yo-Yo IR2 after intense arm exercise. Where analyses revealed a significant main effect, the origin of this effect was determined by Bonferroni adjusted post hoc paired *t*-tests. Differences in plasma [glucose], [lactate], HR and RPE between the three conditions (PLA, CAF and NaHCO_3_) were analysed using multiple separate two-way repeated measures ANOVAs (supplement x time). Significant main effects were further analysed by Bonferroni adjusted post hoc paired *t*-tests. All repeated measures data was checked for the assumption of sphericity using Mauchly’s test. All data were analysed using the statistical software package SPSS (version 20). Statistical significance was accepted at *P* < 0.05. Results are presented as means ± SD.

## Results

### Performance

**Figure 1:**
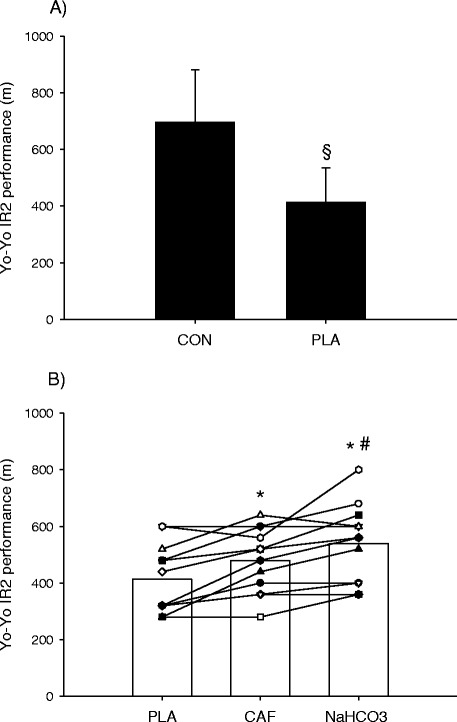
**Yo-Yo Intermittent Recovery level 2 test (Yo-Yo IR2) performance in control (CON) and placebo (PLA) trials (A) and individual Yo-Yo IR2 performance during PLA and after caffeine (CAF) and sodium bicarbonate (NaHCO**_**3**_ **) supplementation (B) (n = 12).** §: Denotes a significant difference from CON. *Denotes a significant difference from PLA. # Denotes a significant difference from CAF. Significance level P < 0.05.

Yo-Yo IR2 performance was reduced (P < 0.05) by 41% when the test was preceded by an intense intermittent arm cranking (CON: 696 ± 185 m vs. PLA: 413 ± 121 m; *P* < 0.01, Figure 1A). However, after CAF and NaHCO_3_ supplementation Yo-Yo IR2 performance was 14 and 23% higher (P < 0.05) compared to PLA (480 ± 113 and 540 ± 138 m, respectively), with a greater (P < 0.05) improvement in NaHCO_3_ than CAF (Figure 1B).

### Blood metabolites

**Figure 2:**
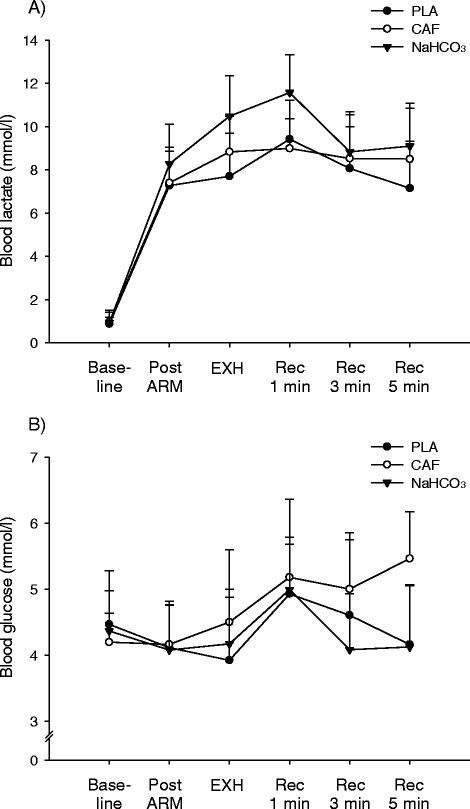
**Capillary blood lactate (A) and glucose (B) concentrations before, during and after the Yo-Yo IR2 test protocol (n = 12) in the placebo (PLA), caffeine (CAF) and sodium bicarbonate (NaHCO**_**3**_ **) trials.**

Baseline blood [Lactate] was similar (0.9 ± 0.3, 1.0 ± 0.5 and 0.9 ± 0.6 mmol.L^−1^; P > 0.05) for PLA, CAF and NaHCO_3_, respectively, but increased (*P* < 0.01) post arm crank (7.3 ± 1.8, 7.4 ± 1.5 and 8.3 ± 1.8 mmol.L^−1^, respectively, Figure 2A) with no significant differences between the three trials, although there was a trend (*P* = 0.09) for higher blood [lactate] for the NaHCO_3_ trial. Blood [lactate] did not change (*P* > 0.05) during Yo-Yo IR2 for PLA at exhaustion (7.7 ± 2.0 mmol.L^−1^), but rose (*P* < 0.05) for both CAF and NaHCO_3_ (8.8 ± 1.7 vs. 10.5 ± 1.9 mmol.L^−1^, respectively, Figure 2A). Between group comparisons revealed that blood [lactate] values were similar between PLA and CAF, but higher (*P* < 0.01) for the NaHCO_3_ trial compared to PLA and CAF trials at exhaustion (Figure 2A). At 1 min post exhaustion PLA blood [lactate] rose (9.4 ± 1.8 mmol.L^−1^; *P* < 0.05) compared to exhaustion whereas CAF and NaHCO3 remained unchanged (*P* > 0.05) and blood [lactate] in the NaCHO_3_ trial still remained higher (11.6 ± 1.7 mmol.L^−1^; *P* < 0.01) when compared to PLA and CAF. At 3 min post Yo-Yo IR2 [lactate] was not different between groups (8.1 ± 1.9, 8.5 ± 2.2 and 8.8 ± 1.7 mmol.L^−1^; *P* > 0.05) PLA, CAF and NaHCO_3_, respectively. At 5 min into recovery Yo-Yo IR2 blood [lactate] was reduced in the PLA trial when compared to CAF and NaHCO_3_ (7.2 ± 2.2 vs. 8.5 ± 2.3 and 9.1 ± 1.9 mmol.L^−1^; *P* < 0.05, respectively) whereas CAF and NaCHO_3_ were similar at this time point (*P* > 0.05).

Baseline blood [glucose] was not significantly different between trials (4.5 ± 0.5 vs. 4.2 ± 0.4 vs. 4.4 ± 0.9 mmol.L^−1^; *P* > 0.05, Figure 2B) for PLA, CAF and NaHCO_3_, respectively. For all trials at Yo-Yo IR2 exhaustion there was no difference (*P* > 0.05) in blood [glucose] within and between the supplements (3.9 ± 0.9 vs. 4.5 ± 1.1 vs. 4.2 ± 0.8 mmol.L^−1^, respectively, Figure 2B). In the PLA and NaHCO_3_ trials blood [glucose] rose (P < 0.05) between exhaustion and 1 min post Yo-Yo IR2 (4.9 ± 0.9 and 4.9 ± 0.7 mmol.L^−1^). In the CAF trial blood [glucose] rose by 30% at the end of the protocol (Figure 2B), compared to the PLA (*P* < 0.05). However, no differences (*P* > 0.05) were in [glucose] between PLA and NaHCO_3_ at any protocol time points (Figure 2B). Between group comparisons revealed that at 5 min post Yo-Yo IR2 blood [glucose] in the CAF trial was greater (*P* < 0.05) compared to the PLA and NaHCO_3_ (5.5 ± 0.7 vs. 4.2 ± 0.9 and 4.1 ± 0.9 mmol.L^−1^, respectively).

### Rating of perceived exertion and heart rate loading

**Figure 3:**
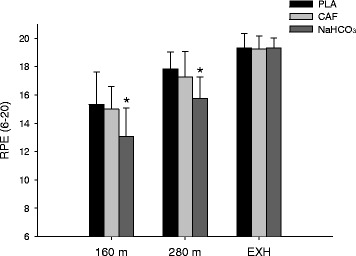
**Rating of Perceived Exertion (RPE) at 160 and 280 m during the Yo-Yo IR2 test, as well as at exhaustion in PLA, CAF and NaHCO**_**3**_ **.** *Denotes a significant difference from PLA. Significance level P < 0.05.

Between group comparisons revealed that RPE was reduced (*P* < 0.05) in the NaHCO_3_ trials at both 160 m and 280 m (160 m: 15.3 ± 2.3 and 15.0 ± 1.6 vs. 13.1 ± 2.0), (280 m: 17.8 ± 1.2 and 17.3 ± 1.8 vs. 15.8 ± 1.5) for PLA, CAF and NaHCO_3_, respectively (Figure 3). No differences (*P* > 0.05) were observed at Yo-Yo IR2 exhaustion (19.3 ± 1.0, 19.3 ± 0.9 and 19.3 ± 0.7 in PLA, CAF and NaHCO_3_, respectively, Figure 3). Arm exercise caused a rise (*P* < 0.01) in heart rate during all trials (179 ± 7, 178 ± 8 and 177 ± 9 bts.min^−1^) for PLA, CAF and NaHCO_3_, respectively. At Yo-Yo IR2 exhaustion a further rise (*P* < 0.05) in heart rate for CAF and NaHCO_3_ was observed (187 ± 7 and 186 ± 7 bts.min^−1^, respectively). Comparisons between trials revealed that there was no difference between PLA, CAF and NaHCO_3_ at any time points during the testing protocol.

## Discussion

The principle findings of the present study were that Yo-Yo IR2 performance after intense arm cranking exercise was markedly reduced compared to a control trial, while both caffeine and NaHCO_3_ intake improved fatigue resistance during the Yo-Yo IR2 test. NaHCO_3_ intake elicited a further ergogenic effect on Yo-Yo IR2 performance when performed after intense arm cranking exercise compared to that of caffeine. The present study is the first to report performance effects of caffeine and NaHCO_3_ intake using high-intensity intermittent running exercise preceded by intense arm cranking exercise and to demonstrate additional performance and perceptual benefits of NaHCO_3_ beyond that of caffeine.

The reduction in high-intensity running performance when performed after intense arm cranking exercise is consistent with previous research studying isolated muscle performance [14,15]. In the present study, Yo-Yo IR2 performance was reduced by 41% when preceded by arm exercise, which is of a similar magnitude as the performance decrements of 35% and ~48%, respectively, observed during intense exhaustive one-legged knee extensor exercise performed after intense arm cranking [14,15]. These two studies also showed that the arm exercise resulted in metabolic acidosis with arterial blood lactate being elevated by ~11-fold resting levels, as well as higher leg muscle lactate and H^+^ concentrations [14] and elevated accumulation rate of leg muscle interstitial [K^+^] [15]. In the present study capillary blood lactate was elevated ~8-fold after the arm cranking protocol, confirming that the metabolic environment was markedly altered prior to the Yo-Yo IR2 test compared to baseline conditions.

Yo-Yo IR2 performance was improved by 23% after NaHCO_3_ supplementation, which is supported by others [9,10]. Indeed performance enhancement in the final sprints of a repeated sprint test after NaHCO_3_ intake has been reported [10], indicating an ergogenic effect of NaHCO_3_ on fatigue resistance during high intensity exercise conditions performed under metabolic stress. However, some studies applying intense exercise protocols report no beneficial effect of NaHCO_3_ supplementation (for review see Carr et al. [9]). In the present study intense arm cranking markedly elevated blood lactate, confirming findings of an increased leg muscle lactate and H^+^ concentration following the same arm protocol [14]. Part of the longer exercise time level in the NaHCO_3_-trial may be explained by an elevated NaHCO_3_ induced buffer capacity in the blood, which will increase the muscle-to-blood H^+^ and lactate gradient. Since the monocarboxylate transporters (MCT) are gradient dependent [17], this will increase the removal of H^+^ and lactate ions from the leg muscles before and during the Yo-Yo IR2 test. This may reduce the degree of intramuscular acidification and corresponding fatigue development [1]. The higher blood lactate levels were observed in the NaHCO_3_-trial, may also partly be a direct consequence of the greater fatigue resistance, and thereby higher glycolytic contribution and concomitant muscle lactate production compared to the caffeine and placebo trials. A limitation with the present study is that blood was not drawn during the Yo-Yo IR2 test, which have been done on other comparable studies [5,13], so we are unable to compare the three intervention trial at fixed time points due to the different exercise times.

The intake of NaHCO_3_ may also affect the activity of sarcolemmal ion transporters. For example elevated extracellular Na^+^ concentration may stimulate the Na^+^/H^+^ exchangers [18] and thereby increase the efflux of hydrogen ions from the exercising muscles and attenuate intramuscular acidification. In a study by Street et al. [19] muscle interstitial pH decreased gradually during graded exercise comparable to the Yo-Yo IR2 test. However, in a follow-up study sodium citrate was ingested prior to intense exercise, which on one hand elevated plasma HCO_3_^−^ and lowered the accumulation of muscle interstitial H^+^ [18]. Finally, the accumulation rate of interstitial K^+^ was significantly reduced, which was suggested to relate to less opening probability of the K_ATP_ channels, which tend to open during intracellular acidification [20]. In addition, the elevated systemic Na^+^ may directly stimulate the activity in the Na^+^-K^+^ ATPase [1,18]. Thus, the intake of NaHCO_3_ may have elevated fatigue resistance by inducing maintenance of a more optimum intracellular pH [21] and/or by enhancing Na^+^/K^+^ pump activity and potentially Na^+^/K^+^/2Cl^−^ co-transporter activity, contributing to lower muscle interstitial [K^+^] preserving sarcolemma excitability, allowing enhanced high intensity repeated muscular performance [1].

RPE was lower in NaHCO_3_-trial during the Yo-Yo IR2 test, which may suggest that centrally mediated mechanisms were affected. The participants experienced less exertion during the Yo-Yo IR2 in the NaHCO_3_-trial, and that although distance covered before exhaustion was increased the RPE values remained the same as the caffeine and placebo trials at the point of fatigue. Thus, a greater distance could be covered yet reporting an equal level of perceived fatigue at exhaustion. Peripheral changes may cause modulation of neural strategies via group III and IV muscle afferents which are widely distributed through the muscle and are responsive to chemical stimuli such as altered H^+^ and K^+^ [22]. Different mechanisms have been proposed by which such peripheral inputs might lead to modulation of motor neuron firing rates and muscle function including facilitation or inhibition of spinal reflexes, pre-synaptic inhibition of the group Ia afferents thus reducing inhibitory input into the motoneurons, and altered supraspinal drive [22]. Thus, part of the improved performance after NaHCO_3_ treatment may relate to less negative feedback from the muscle and thereby less effect on the descending drive to the motoneurons [22,23].

In the present study caffeine increased Yo-Yo IR2 performance by 14% compared to the placebo-trial, which is in line with other findings [3,4]. Caffeine has multiple physiological effects that may promote fatigue resistance during intense exercise, such as elevating the catecholamine levels [24] and reducing muscle interstitial K^+^ accumulation [5]. Peak blood glucose concentrations were higher in the caffeine-trial than placebo, which is in accordance with findings by others [5], indicating an elevated catecholamine response, which may facilitate the Na^+^-K^+^ ATPase activity [25]. The arm protocol has been shown to increase the accumulation of interstitial potassium during leg exercise [15], and caffeine may reduce this effect by increasing the pump activity in the Na^+^-K^+^ APTase directly or via an elevated catecholamine response [5,25]. Finally caffeine may have an effect on central mechanisms associated with fatigue [23,26], however, the RPE rating were not different between the caffeine and placebo trials in the present study. Caffeine is well-known to affect the central nervous system response mediated via antagonism of adenosine receptors, which dampens pain perception and attenuates fatigue [27]. However, during high-intensity intermittent exercise caffeine-induced effects on RPE appear to be negated [6], which is supported by this study.

Intriguingly, there were no differences in HR between the trials, suggesting that the aerobic demands were similar, yet participant’s perception of fatigue was attenuated during the NaHCO_3_-trial, indicating that NaHCO_3_ in contrast to caffeine might impact upon central fatigue as well as peripheral fatigue.

The possibility of a ‘high-responders’ and ‘low-responders’ phenomena for both caffeine and NaHCO_3_ has been previously reported [27-29]. This effect was apparent in the present study, whereby three subjects and one subject, respectively, after caffeine and NaHCO_3_ intake, showed no improvement compared to PLA. Moreover five of the subjects reported experiencing some form of gastrointestinal discomfort after sodium bicarbonate intake; however this was apparently not detrimental to exercise performance.

## Conclusions

The present study demonstrates that high-intensity intermittent exercise performance is impaired when preceded by intense arm exercise. However, fatigue resistance can be markedly improved by caffeine and NaHCO_3_ administration 70–90 min prior to exercise. Moreover, for the first time NaHCO_3_ has been shown to elicit an improvement above that of caffeine in a sport-specific high-intensity intermittent test with concomitant reductions in RPE and higher blood lactate levels. This study suggests that caffeine and NaHCO_3_ might be effective ergogenic aids for intermittent high-intensity exercise performance in sub-elite team sport athletes.
